# Liver-specific overexpression of LPCAT3 reduces postprandial hyperglycemia and improves lipoprotein metabolic profile in mice

**DOI:** 10.1038/nutd.2016.12

**Published:** 2016-04-25

**Authors:** J G Cash, D Y Hui

**Affiliations:** 1 Department of Pathology and Laboratory Medicine, Metabolic Disease Research Center, University of Cincinnati College of Medicine, Cincinnati, OH, USA

## Abstract

Previous studies have shown that group 1B phospholipase A_2_-mediated absorption of lysophospholipids inhibits hepatic fatty acid β-oxidation and contributes directly to postprandial hyperglycemia and hyperlipidemia, leading to increased risk of cardiometabolic disease. The current study tested the possibility that increased expression of lysophosphatidylcholine acyltransferase-3 (LPCAT3), an enzyme that converts lysophosphatidylcholine to phosphatidylcholine in the liver, may alleviate the adverse effects of lysophospholipids absorbed after a lipid-glucose mixed meal. The injection of an adenovirus vector harboring the human *LPCAT3* gene into C57BL/6 mice increased hepatic LPCAT3 expression fivefold compared with mice injected with a control LacZ adenovirus. Postprandial glucose tolerance tests after feeding these animals with a bolus lipid-glucose mixed meal revealed that LPCAT3 overexpression improved postprandial hyperglycemia and glucose tolerance compared with control mice with LacZ adenovirus injection. Mice with LPCAT3 overexpression also showed reduced very low density lipoprotein production and displayed elevated levels of the metabolic- and cardiovascular-protective large apoE-rich high density lipoproteins in plasma. The mechanism underlying the metabolic benefits of LPCAT3 overexpression was shown to be due to the alleviation of lysophospholipid inhibition of fatty acid β-oxidation in hepatocytes. Taken together, these results suggest that specific LPCAT3 induction in the liver may be a viable strategy for cardiometabolic disease intervention.

## Introduction

The postprandial period immediately after meal feeding is the most at risk time for cardiovascular events as well as promoting tissue insulin resistance and diabetes.^1, 2, 3^ The mechanisms underlying postprandial dysregulation of lipid and glucose metabolism have been studied extensively. One enzyme that has a major role in diet-induced cardiometabolic diseases is the group 1B phospholipase A_2_ (PLA2G1B) that catalyzes fat digestion in the intestinal lumen after meal feeding. In humans, polymorphism in the *PLA2G1B* gene is a determinant of central obesity.^4^ In rodent models, both chemical inhibition and genetic inactivation of PLA2G1B are protective against obesity and hyperglycemia in response to chronic feeding of high fat/high carbohydrate diet.^5, 6^

The contribution of PLA2G1B toward diet-induced hyperlipidemia and hyperglycemia appears to occur predominantly during the postprandial period due to the amount of lysophospholipids generated and absorbed during the digestion process.^7, 8^ Lysophospholipids influence lipid and glucose homeostasis by reducing mitochondrial fatty acid oxidation in the liver.^8, 9^ Another enzyme that modulates lysophospholipid availability and metabolism in the liver is lysophosphatidylcholine acyltransferase-3 (LPCAT3), a protein that catalyzes the reacylation of lysophospholipids to phospholipids. The knockdown of LPCAT3 activity has been shown to increase intracellular lysophospholipid levels and promote very low density lipoprotein (VLDL) secretion in hepatocytes.^10^ These observations suggested that increasing LPCAT3 activity in the liver may lower lysophospholipid levels and confer metabolic benefits. In support of this hypothesis is the recent study indicating that LPCAT3 is an important mediator of liver X receptor effects on metabolism.^11^ These investigators showed that LPCAT3 expression in the liver improves insulin sensitivity in *ob/ob* and *db/db* mice due to changes in membrane composition that ameliorate ER stress in response to saturated fatty acids. Whether the metabolic benefits of LPCAT3 are restricted to improvement in response to saturated fatty acids remains known. In particular, these studies did not evaluate the consequence of LPCAT3 overexpression on postprandial lipid and glucose homeostasis in wild-type mice, nor determined whether the mechanism may be related to alleviate lysophospholipid inhibition of fatty acid β-oxidation. Clarification of these issues is necessary to substantiate the hypothesis that specific elevation of LPCAT3 expression/activity may be a viable option for metabolic disease therapy without the adverse effects associated with transcription factor activation. The current study utilizes adenovirus-mediated LPCAT3 gene transfer approach to test this hypothesis.

## Materials and methods

### Experimental animals

Male C57BL/6J mice (10–15 weeks old) (Jackson Laboratory, Bar Harbor, ME, USA) were maintained in an environmentally-controlled room and fed a rodent chow (LM485; Harlan-Teklad, Madison, WI, USA) with free access to water. All animal protocols used in this study were approved by the institutional animal care and use committee at the University of Cincinnati, and in compliance with the principal of laboratory animal care policy of the National Institutes of Health.

### Adenovirus preparation and experimentation

Adenovirus particles containing cDNA for human *LPCAT3* and bacterial *LacZ* were purchased from Applied Biological Materials (Richmond, BC, Canada) and amplified in HEK293 cells. The supernatant containing 4 × 10^9^ viral particles per ml was dialyzed into a solution of 10 mm Tris-HCl, pH 8.0, 2 mm MgCl_2_ and 5% sucrose. One hundred microliters (4 × 10^8^ PFU) of the adenovirus encoding human *LPCAT3* or the control LacZ gene was injected intravenously into wild-type C57BL/6J mice. The animals were not randomized before assignment for *LPCAT3* or *LacZ* adenovirus injection. Expression level of LPCAT3 in the liver was assessed by western blot analysis with anti-LPCAT3 (cat. #sc161831; Santa Cruz Biotechnology, San Cruz, CA, USA).

### Glucose tolerance test

Glucose tolerance was monitored in mice after an overnight fast. On the day of experiments, the mice were fed 4 ml kg^−1^ body weight of a hypercaloric lipid-glucose mixed meal containing 2.6 mm egg phosphatidylcholine, 13.33 mm triolein and 2.6 mm cholesterol in a saline solution containing 50% glucose. Blood was obtained before and at different times after administration of the test meal. Blood glucose levels were measured with an Accu-Chek Glucometer (Roche Applied Science, Indianapolis, IN, USA).

### Lipid and lipoprotein determinations

Liver and plasma triglyceride and cholesterol levels were determined using the Infinity Triglyceride and Cholesterol Assay Kits (Thermo Fisher Scientific, Middletown, NJ, USA). Plasma lipoprotein profiles were determined from 200 μl of plasma by fast performance liquid chromatography on a Superose 6 HR 10/30 column (Amersham Biosciences, Piscataway, NJ, USA), and 0.5 ml fractions were collected for cholesterol determinations. Western blot analysis was performed on selected fractions with anti-mouse apoA-I (cat. #ab7614; Abcam Antibodies, Cambridge, MA, USA) and apoE antibodies (cat. #sc6384; Santa Cruz Biotechnology) as described previously.^12^ Hepatic lysophosphatidylcholine (LPC) levels were measured in liver homogenates by modification^7^ of the enzymatic assay procedure originally described by Kishimoto *et al.*^13^ Hepatic secretion of high density lipoproteins (HDL) was assessed *in vitro* using primary hepatocytes isolated from mice 2 days after injection with adenovirus encoding either LacZ or LPCAT3 using the exact procedure that we have described previously.^12^

### Cellular oxygen consumption

Primary hepatocytes were prepared from anesthetized mice after isoflurane inhalation by perfusion with 100 U ml^−1^ collagenase.^12^ The hepatocytes were plated on a collagen-coated 24-well Seahorse plate (Seahorse Bioscience, North Billerica, MA, USA) at a density of 12 500 cells per well before incubation with oleate and LPC, 10 μg ml^−1^ oligomycin, 3 μm carbonyl cyanide 4-(trigluoromethoxy)phenyhydrazone (FCCP) and 4 μm antimycin A/1 μm rotenone to measure oxidation changes and construct mitochondrial bioenergetics profiles.^9^ Basal oxygen consumption rate (OCR) was established before oleate and LPC addition, and the influence of oleate and LPC on OCR was reported as changes from basal OCR.^9^

### Statistics

All results are expressed as means±s.d. with sample size of *N*=6 or *N*=9 as indicated in the figure legends for each experiment. Sample size was chosen based on previous results indicating that *N*=6 is sufficient to yield statistical significant differences in the effect of PLA2G1B-generated lysophospholipids in postprandial glucose tolerance.^7^ Data were analyzed in an unblinded manner. Statistical analyses were performed using two-way ANOVA for repeated measurements followed by Bonferroni *post hoc* analysis or unpaired two-tailed Student's *t*-test assuming equal variance for single point measurements. Differences were considered as significant at *P*<0.05 based on ANOVA with Bonferroni *post hoc* test or Student's *t-*test.

## Results and Discussion

**Figure 1:**
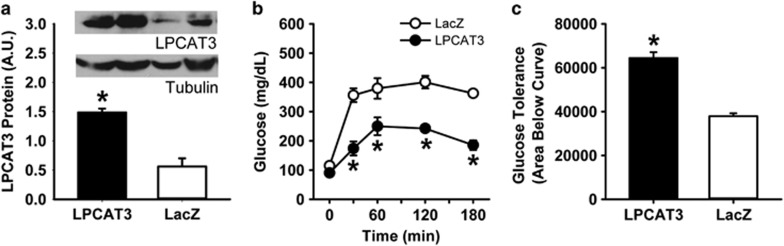
Improved glucose tolerance in mice with adenovirus-mediated overexpression of LPCAT3. C57BL/6 mice were injected with adenovirus encoding either human LPCAT3 (filled bars and symbols) or LacZ (open bars and symbols). (**a**) Western blot analysis of LPCAT3 protein levels 2 days after adenovirus injection. The inset shows representative western blot images of LPCAT3 levels relative to tubulin levels in the respective livers. The data represent mean±s.d. from three separate experiments (total *N*=6). (**b**) Glucose tolerance was performed 2 days after injection. Mice were fasted overnight and blood glucose levels were measured before and after feeding a bolus lipid-glucose mixed meal. (**c**) Area under the curve analysis of data from the glucose tolerance tests. The glucose tolerance data represent mean±s.d. from two separate experiments each performed with three mice in each group (total *N*=6). The data were analyzed by *t*-test (**a**, **c**) or ANOVA (**b**) with * denotes differences from the LacZ group at *P*<0.05.

The total (human+mouse) LPCAT3 protein levels in the livers of mice injected with *LPCAT3* encoding adenovirus were ~5-fold higher than mice receiving the LacZ adenovirus (Figure 1a). Postprandial glucose tolerance tests after feeding these animals with a bolus lipid-glucose mixed meal revealed glucose intolerance in LacZ adenovirus recipient mice indicative of impaired glucose homeostasis with acute adenovirus infection.^14^ Interestingly, LPCAT3 overexpression recapitulated the phenotype observed in *Pla2g1b*^*−/−*^ mice,^7^ with improved postprandial hyperglycemia and glucose tolerance compared with the control mice with LacZ adenovirus injection (Figures 1b and c).

**Figure 2:**
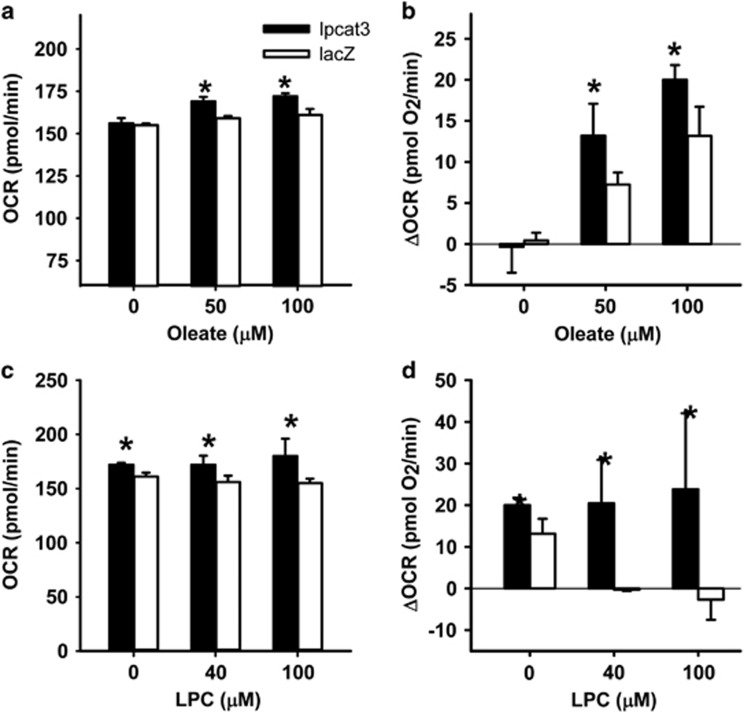
Improved hepatic fatty acid β-oxidation in mice with adenovirus-mediated overexpression of LPCAT3. Primary hepatocytes were isolated from mice 2 days after injection of adenovirus encoding either LPCAT3 or LacZ gene. (**a**) OCRs were measured after incubation with or without oleate addition. (**b**) Fatty acid β-oxidation rates reported as OCR changes (ΔOCR) compared with cells incubated in the absence of oleate. (**c**) LPC inhibition of oxygen consumption by control (LacZ) and LPCAT3 overexpressing hepatocytes (LPCAT3) induced by 100 μm oleate. (**d**) Analysis of the data in (**c**), which showed that LPCAT3 overexpression suppresses LPC-induced OCR change. The data represent mean±s.d. from three separate experiments, each performed with triplicate determinations. * denotes differences from the LacZ control group at *P*<0.05.

The improved postprandial glucose tolerance in *Pla2g1b*^*−/−*^ mice is due to elevated fatty acid β-oxidation in the liver of these animals.^7, 9^ To test the possibility that the improved postprandial glucose tolerance observed in LPCAT3 overexpressing mice was also due to an increase in hepatic fatty acid β-oxidation, hepatocytes isolated from mice after injection with LPCAT3 or LacZ adenoviruses were incubated *in vitro* with oleic acid in the presence or absence of LPC. Mitochondrial fatty acid β-oxidation rates, as measured by cellular OCRs in the presence or absence of fatty acids,^9^ were significantly higher with LPCAT3 overexpression compared with controls (Figures 2a and b), presumably through reducing intracellular levels of LPC that has been shown previously to cause mitochondrial dysfunction.^9^ Indeed, OCR in LPCAT3 overexpressing hepatocytes was also less sensitive to suppression by exogenously supplied LPC (Figures 2c and d). These data are consistent with the interpretation that overexpression of LPCAT3 increases the utilization of lysophospholipid for phospholipid synthesis, thereby alleviating lysophospholipid inhibition of fatty acid oxidation and improving glucose tolerance.

**Figure 3:**
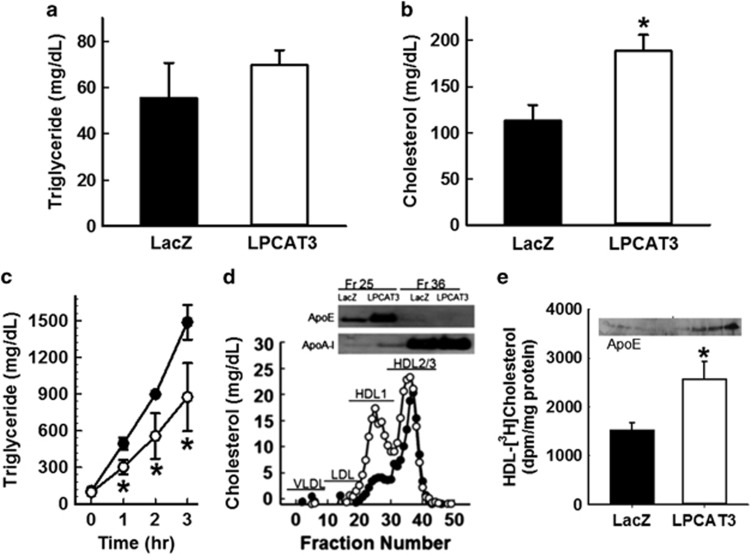
Adenovirus-mediated LPCAT3 overexpression improves plasma lipoprotein profile in mice. Fasting plasma levels of triglyceride (**a**) and cholesterol (**b**) in male C57BL/6 mice 2 days after injection with adenovirus encoding either human LPCAT3 (open bars) or LacZ (filled bars). (**c**) Results of VLDL synthesis by LacZ control (filled symbols) or LPCAT3 overexpressing (open symbols) mice as measured by plasma triglyceride measurements after injection of the lipolytic inhibitor Poloxamer 407 are shown. (**d**) Representative FPLC plasma lipoprotein profile from LacZ control (filled symbols) and LPCAT3 overexpressing mice (open symbols) is shown. Fractions containing VLDL, LDL, HDL_1_ and HDL_2_/HDL_3_ were identified by comparison with controls. The inset shows western blots of apoE and apoA-I in the HDL_1_ fraction (Fr. 25) and the HDL_2_/HDL_3_ fraction (Fr. 36). (**e**) Secretion of HDL cholesterol from [^3^H]cholesterol-loaded hepatocytes with LacZ adenovirus (filled bar) and LPCAT3 adenovirus (open bar) is shown. The inset shows apoE enrichment in HDL particles secreted by LPCAT3 overexpressing hepatocytes. The data representing mean±s.d. from three separate experiments, each performed with triplicate determinations, were analyzed by *t*-test (**a**, **b**, **e**) or ANOVA (**c**) with * denotes differences from the LacZ control group at *P*<0.05.

Hepatic LPCAT3 overexpression may also alter lipoprotein metabolism. Interestingly, while no significant differences were observed in fasting plasma triglyceride levels between mice receiving LPCAT3 or LacZ adenoviruses (Figure 3a), fasting plasma cholesterol levels were significantly increased in mice after LPCAT3 adenovirus injection (Figure 3b). The mechanism responsible for the LPCAT3-induced plasma cholesterol levels was explored by determining hepatic VLDL production as measured by plasma triglyceride levels from 0 to 3 h after injection of Poloxamer 407 (1 g kg^−1^ b.w.) to block triglyceride-rich lipoprotein clearance.^15^ Results showed that hepatic LPCAT3 overexpression reduced VLDL production (Figure 3c), which are consistent with results of opposing studies showing that LPCAT3 knockout promotes VLDL secretion.^10^ Thus, the increase in fasting plasma cholesterol levels in LPCAT3 overexpressing mice was not due to increased production of apoB-containing lipoproteins. Analysis of fasting plasma lipoprotein profile in these animals revealed a striking increase in large HDL particles in mice with LPCAT3 adenovirus injection compared with the LacZ adenovirus-injected mice (Figure 3d). Analysis of proteins associated with these large HDL confirmed the absence of apoB and that these are apoE-rich and apoA-I-poor HDL_1_ (Figure 3d, inset). These data suggested that LPCAT3 overexpression may increase HDL secretion. This hypothesis was supported by *in vitro* experiments showing that primary hepatocytes prepared from LPCAT3 adenovirus-transduced mice displayed elevated secretion of HDL cholesterol compared with hepatocytes from LacZ adenovirus recipient mice (Figure 3e). Moreover, the HDL secreted by LPCAT3 overexpressing hepatocytes was also enriched with apoE (Figure 3e, inset). These apoE-rich large HDL are similar in characteristics as those reported previously to be induced by liver X receptor agonists,^16^ and are likely synthesized through mechanisms distinct from synthesis of normal apoA-I HDL.^17^ Importantly, these apoE-rich HDL are metabolic and cardiovascular protective^18^ and are found to be depleted in subjects with type 2 diabetes.^19^

**Figure 4:**
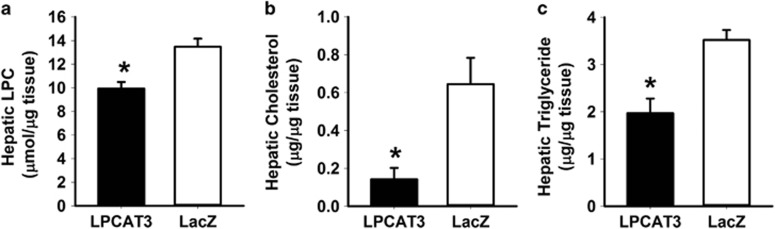
Adenovirus-mediated LPCAT3 overexpression reduces postprandial lipid levels in liver. Adenovirus LPCAT3 (filled bars) or LacZ (open bars) recipient mice were fed a bolus lipid meal 2 days after adenovirus injection. Hepatic levels of LPC (**a**), cholesterol (**b**) and triglyceride (**c**) were determined 3 h after lipid meal feeding. The data representing mean±s.d. for triplicate determinations were analyzed by *t-*test with * denotes differences from the LacZ control group at *P*<0.05.

The metabolic benefits of LPCAT3 overexpression are similar to those observed with *Pla2g1b*^−^^*/−*^ mice, suggesting that the mechanism is likely mediated via reduction of LPC levels in the liver. Direct LPC in the liver confirmed the reduction of this bioactive metabolite in the livers of LPCAT3 adenovirus recipient mice after feeding a lipid-rich meal compared with that observed in LacZ adenovirus recipient mice (Figure 4a). Importantly, postprandial hepatic triglyceride and cholesterol levels were also reduced in mice with LPCAT3 overexpression (Figures 4b and c). Taken together, these data are consistent with the interpretation that increased expression of LPCAT3 reduces the postprandial LPC levels in the liver and consequentially enhances mitochondrial fatty acid oxidation, improves postprandial glucose tolerance and promotes a favorable lipoprotein profile without the adverse VLDL synthesis promoting effects of liver X receptor agonists. Hence, strategies that lead to direct induction of LPCAT3 expression in the liver may be explored to suppress diet-induced cardiometabolic diseases.
